# Rational syntheses of core-shell Fe*_x_*@Pt nanoparticles for the study of electrocatalytic oxygen reduction reaction

**DOI:** 10.1038/srep02872

**Published:** 2013-10-07

**Authors:** Ji-Hoon Jang, Eunjik Lee, Jinwoo Park, Gunn Kim, Suklyun Hong, Young-Uk Kwon

**Affiliations:** 1 Department of Chemistry, HRD Center for Creative Convergence Chemical Sciences, SAINT/Center for Human Interface Nano Technology, Sungkyunkwan University, Suwon 440-746, Republic of Korea; 2 Department of Physics and Graphene Research Institute Sejong University, Seoul 143-747, Republic of Korea

## Abstract

We report on the syntheses of core-shell Fe*_x_*@Pt (*x* = 0.4–1.2) nanoparticles (NPs) with Pt-shell thickness systematically controlled while the overall particle size is constant. The syntheses were achieved via one-pot ultrasound-assisted polyol synthesis (UPS) reactions. Fe_1.2_@Pt showed a record-breaking high core-element content (55 at%) of core-shell NPs. Based on observations from a series of control experiments, we propose a mechanism of the NPs' formation that enables control of shell thickness in UPS reactions. Fe*_x_*@Pt NPs showed drastic enhancements in mass and specific activity for oxygen reduction reaction (ORR) and significantly enhanced durability compared to commercial Pt NPs. Fe*_x_*@Pt with a 1 (monolayer) ML Pt shell showed the highest activity. The *ab initio* density functional theory calculations on the binding energies of oxygen species on the surfaces of Fe*_x_*@Pt NPs showed that the 1 ML case is most favourable for the ORR, and in good agreement with the experimental results.

Core-shell nanoparticles (NPs) have attracted enormous interest for their variously tunable physicochemical features^1^,^2^,^3^,^4^. Especially in polymer electrolyte membrane fuel cell (PEMFC) applications, NPs composed of a transition metal (M) core and a Pt or Pd shell have several important benefits as electrocatalysts^3^,^4^,^5^,^6^,^7^,^8^. Such M@Pt core-shell NPs were reported to have significantly increased electrocatalytic efficiency and durability for the oxygen reduction reaction (ORR) on the cathodes of PEMFCs.

In the ORR, the release of oxygenated species, such as O and OH, is the rate-limiting step on the majority (111) facets of Pt NPs^9^,^10^,^11^. Enhancement of the ORR by core-shell NPs has been explained as an ensemble effect, a modification of the electronic structure, and a lattice strain effect^12^,^13^,^14^. Therefore, the key for the successful development of core-shell NPs for enhanced ORR depends on optimal design of the NPs, including the choice of the core element and control of the Pt shell thickness, along with other factors. As for the shell thickness, it is reported that bi- or tri-monolayers of Pt are maximal when the core is composed of Pd, Ru or Ni^6^,^15^,^16^. No further studies have been reported for other core elements, and it is possible that the maximal shell thickness is core-element specific.

Several different methods to synthesise core-shell NPs have been reported. The most widely used approach is the underpotential deposition (UPD) method^17^,^18^. In this method, a few layers of Cu and/or Pb are deposited on the “core” NPs electrochemically, and then the Cu/Pb adlayers are replaced with noble metals, such as Pt or Au, by galvanic displacement. The UPD method can form fine-structured core-shell NPs with controlled shell thicknesses. However, generally, the sample scale is restricted to be very small because the core NPs must be immobilised on the electrode for the deposition of Cu/Pb, unless the electrode has a very large surface area^19^. Furthermore, the whole process is complicated, involving multiple steps. Recently, a method to induce phase segregation within individual NPs by heat-treatment under hydrogen to prepare PdCo@Pd/C electrocatalyst has been reported^20^. Although the process is simplified, this method requires a high temperature, resulting in larger NPs for which the control of shell thickness is not easy. Acid-treatment and voltammetric surface dealloying of alloy NPs can selectively remove non-noble metals on the surface to enrich the surface with noble metal, but these methods also involve heat treatment^8^,^21^.

In this study, we synthesised Fe core-Pt shell (Fe@Pt) NPs with various Fe/Pt ratios (PtFe*_x_*; *x* = 0.4–1.2) by one-step sonochemical reactions. This synthesis does not require any further treatment by heat or acid. The NPs of the four samples are of the same size, but their shell thickness is systematically varied according to the composition *x*. To the best of our knowledge, this is the first study in which NPs of the same size but with different shell thicknesses are directly compared. In addition, there is no precedent of binary core-shell NPs with the non-noble metal content higher than 50%. It was reported that Pt segregation into the shell becomes impossible by heat-treatment when the concentration of non-noble metal is high^22^. In contrast, as is shown below, PtFe_1.2_ has the highest ORR activity among the four samples.

## Results

The PtFe*_x_* samples were prepared via ultrasound-assisted polyol synthesis (UPS) reactions on iron (III) acetylacetonate (Fe(acac)_3_) and platinum (II) acetylacetonate (Pt(acac)_2_). Briefly, Pt(acac)_2_ and Fe(acac)_3_ were sonicated in ethylene glycol (EG) in the presence of Ketjen black as the carbon support. After the reactions, the dark slurries were filtered, washed and finally dried in a vacuum desiccator. The samples are denoted as PtFe_x_/C. We synthesised four samples with *x* = 0.4, 0.7, 1.0 and 1.2.

A UPS reaction is defined as a sonochemical reaction in an ethylene glycol (or other polyol) medium. Previously, we showed that UPS reactions of metal precursors with high enough vapour pressures produce corresponding metal NPs^23^,^24^. For instance, in Ref 23, Co NPs were formed when volatile Co(acac)_2_ was reacted, whereas nonvolatile Co(acet)_2_ or CoCl_2_ did not produce Co NPs. Further, the reaction of Co(acac)_2_ without sonication produced Co-alkoxide instead of Co NPs. In Ref 24, Pd NPs were formed through the UPS reaction of volatile Pd(acac)_2_.

**Figure 1:**
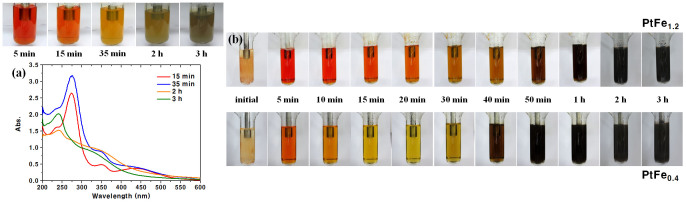
(a) Time variations of appearance and UV-Vis absorption spectra of the UPS reaction of Fe(acac)_3_ and (b) visual observations of the time variations of the UPS reactions for PtFe_1.2_ and PtFe_0.4_.

In the present Fe-Pt system, Fe NPs are much more reactive than Co or Pd NPs, which makes it very hard to produce an XRD pattern or a TEM image of the Fe NPs as direct proof of the formation of Fe NPs. Therefore, we followed the UPS reaction of Fe(acac)_3_ (without Pt(acac)_2_ and carbon support) with UV-Vis absorption spectroscopy (Figure 1(a)). As the reaction proceeds, the deep orange colour of Fe(acac)_3_ is reduced and the solution becomes dark yellow. The spectra show that the peaks of Fe(acac)_3_ have disappeared completely after 2 h, and new peaks appear at 240 and 320 nm, both of which are due to Fe NPs. The 240 nm peak is assigned to the interband transition, and the 320 nm peak to the plasmonic absorption of Fe NPs^25^. Based on these observations, we propose the mechanism of UPS reaction as follows: 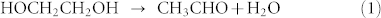

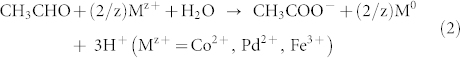
(through the acoustic cavitation process)

For proof of the involvement of EG in UPS reactions, we verified that sonication reactions of Co(acac)_2_ in other solvents, such as hexadecane and diphenyl methane, did not produce Co NPs. Therefore, it is certain that sonication on volatile metal reagents in EG generates conditions in which metal NPs can be formed. In contrast, the non-volatile Pt(acac)_2_ undergoes completely different reaction pathways. We showed previously that the UPS reaction of Pt(acac)_2_ could produce Pt^0^ but with a low efficiency (<10%)^23^. Even in the conventional polyol synthesis of Pt NPs, complete reduction of Pt(II) or Pt(IV) to Pt^0^ requires pH adjustment by adding KOH^26^. Therefore, the low yield of the UPS reaction of Pt(acac)_2_ can be explained in two ways: first, because of its low vapour pressure, Pt(acac)_2_ does not experience the facilitation induced by the acoustic cavitation process. Second, the reaction of Pt(acac)_2_ can still be induced by the heat generated by the sonication. However, the EG solution is acidic due to the protons generated in reaction (2), which suppresses the reduction of Pt(acac)_2_. With these effects combined, the formation of Pt NPs in the present UPS system is effectively suppressed. However, when there are Fe NPs coexisting with Pt(acac)_2_, the following reaction can take place: 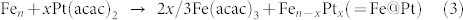
This galvanic replacement reaction can take place without the help of the acoustic cavitation mechanism. Because this reaction occurs on the surface of Fe NPs, the product will be core-shell Fe@Pt NPs.

The Fe_n_ nucleus shown in Figure 1(a) can grow into a larger NP through additional reactions of Pt and Fe atoms. However, the addition mechanisms of Fe and Pt are different. As discussed above, the addition of Pt occurs only through the galvanic replacement with Fe^0^. Therefore, this reaction can occur only when there is an exposed Fe atom on the NP surface. This means that direct deposition of Pt atoms on Pt shells cannot happen. In contrast, the deposition of Fe^0^ atoms can occur on both Fe- and Pt-surfaces because the generation of Fe^0^ is driven by the reaction with EG. The deposition of Fe^0^ atoms on an Fe-surface will effectively increase the core-size (through rearrangement of atoms, which seems to be possible due to the high energy condition produced by the implosion). When Fe^0^ atoms are deposited on a Pt-surface, they can be replaced by Pt^0^ atoms (reaction 3), which can be a way to form an additional layer of Pt on the existing Pt layer.

The relative rates of the depositions of Fe and Pt atoms will affect the shell thickness and the core size (and, thus, the size of the whole NP). The pathways of two extreme cases are schematically drawn in Figure S1 (see Supplementary Information). When the deposition rate of Pt is faster than that of Fe, the growth of the Fe-core will be arrested by the early formation of a Pt shell. Once the Pt shell is completed, additional Fe deposition will occur only on the Pt surface. If there is Pt(acac)_2_ available, such Fe atoms on the Pt-shell will be replaced by Pt atoms, effectively increasing the Pt-shell thickness. As a result, Fe@Pt NPs with small Fe-cores and thick Pt-shells will be produced. Conversely, when the deposition rate of Fe is faster than that of Pt, the Fe-core will grow larger. Since Pt is also deposited and the deposited Pt will form the shell through atomic rearrangement, the surface will eventually be completed by Pt, and the growth of the Fe-core will stop. Once Fe(acac)_3_ is depleted, the surface cannot grow any further. As a result of these processes, the NP will have a monolayer Pt skin.

To support the above explanation, we ran the UPS reactions under the same conditions for PtFe_0.4_/C and PtFe_1.2_/C samples, but without carbon support. The absence of the carbon support enabled us to observe the colour changes as a function of time, as shown in Figure 1(b). Both Fe(acac)_3_ and Pt(acac)_2_ are insoluble to EG at room temperature, and thus the initial solutions are turbid. As the solution is heated by sonication, the reagents are completely dissolved, resulting in clear solutions after 5 min. The deep orange colour of the next stage is mainly due to Fe(acac)_3_; Pt(acac)_2_ is light yellow, which makes its visual observation impossible. Until 40 min for the PtFe_1.2_ reaction and until 20 min for the PtFe_0.4_ reaction, the colour of Fe(acac)_3_ gradually reduces. This corresponds to the consumption of Fe(acac)_3_ to form Fe nuclei. After this, the solution colour becomes brownish and eventually black with a metallic tint, indicating the formation of PtFe_x_ NPs. The onset points of the brown colour and the black colour in the PtFe_0.4_ reaction are about 20 min earlier than in the PtFe_1.2_ reaction, which observation agrees with the mechanism illustrated in Figure S2. Further details in the UPS reaction are explained in SI-1 (see Supplementary Information, Figures S1 and S2).

The compositions of the PtFe_x_ samples were analysed by inductively-coupled-plasma atomic-emission spectrometry (ICP-AES) (see Supplementary Information, Table S1). The compositions of PtFe_x_ NPs are very close to the loaded compositions of the precursors, without significant loss of metals within experimental errors. The compositions of the individual NPs recorded by transmission electron microscopy-energy dispersive spectroscopy (TEM-EDS) are in good agreement with the ICP results (see Supplementary Information, Figure S3). The total metal contents are 24–27 wt% for all of the samples. These results indicate that the UPS method is highly effective in preparing PtFe_x_ NPs with controlled compositions.

**Figure 2:**
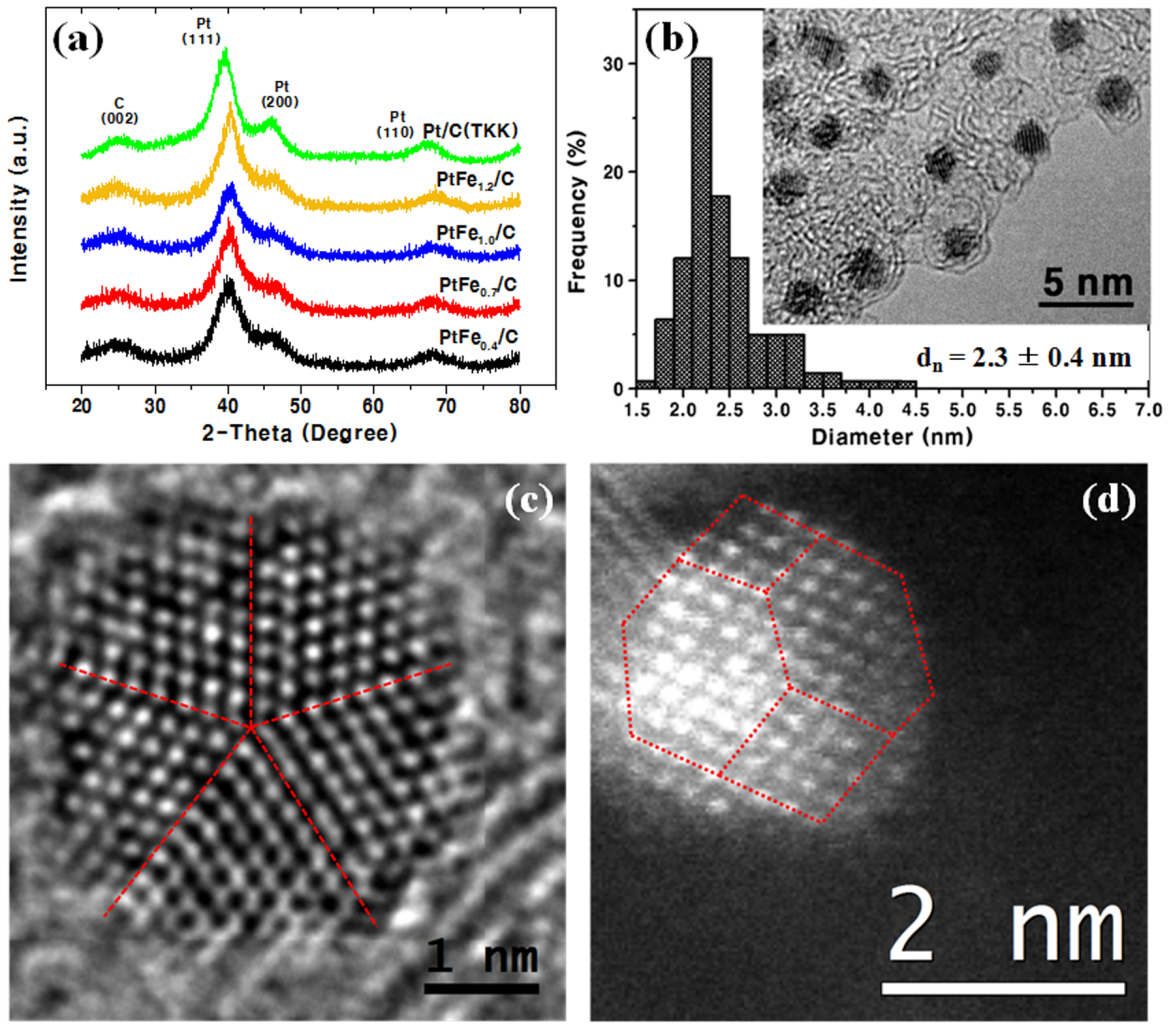
Structural analysis results (a) X-ray diffraction (XRD) patterns of the PtFe_x_/C samples in comparison with commercial Pt/C. (b) Size distribution histogram and transmission electron microscopy (TEM) image, (c) magnified bright field STEM image of the representative PtFe_1.2_ NPs and (d) magnified HAADF-STEM image.

Figure 2 shows the structural analysis results including X-ray diffraction (XRD), TEM and scanning TEM (STEM). The XRD patterns of the PtFe_x_/C samples with the commercial carbon-supported platinum (Pt(TKK), ~2.7 nm) as a reference are shown in Figure 2(a). All of the patterns can be indexed with face-centred cubic (fcc) structure plus the carbon support. In the patterns of the PtFe_x_ samples, the peaks are all shifted to higher angles from the respective peaks of Pt/C. This and the absence of any peak corresponding to iron or iron oxide species indicate that most of the Fe atoms present in our samples are incorporated into the NPs with Pt. From the peak positions, the lattice parameter of the fcc structures are calculated to be: a = ~3.86–3.89 Å, which is smaller than that of Pt, 3.95 Å (Table 1).

The lattice parameters show a gradual decrease as the Pt content decreases. However, they are larger than those reported for Pt-Fe alloy NPs of corresponding compositions, 3.88–3.90 Å^27^, indicating that elemental distributions within the PtFe_x_ NPs are different from the corresponding alloys. From the lattice parameters, the M-M distances are calculated to be 2.73–2.75 Å for PtFe_x_ samples and 2.79 Å for Pt (Table 1). The inner Fe atoms induce compression of the Pt-Pt distance in the shell of Fe@Pt^3^.

Figure 2(b) shows the size distribution of PtFe_1.2_ NPs as a representative example, obtained by measuring the sizes from the TEM image in the inset. Uniform ~2.3-nm-sized NPs with a narrow size distribution (σ = ~0.4 nm) are shown on the carbon support without any agglomeration. The other PtFe_x_ samples show similar behaviours in averaged particle size and size distribution despite the different compositions (see Supplementary Information, Figure S4). The particle sizes agree well with the crystallite sizes calculated with the Scherrer formula.

The particle morphology was investigated by HRTEM images. In the bright-field (BF) image of PtFe_1.2_, we found that most of the NPs were truncated octahedral in shape with minor icosahedral NPs. The image of the latter is shown in Figure 2(c). Unlike icosahedra with five-fold symmetry, truncated octahedral NPs generally do not produce clear BF images, especially when they are very small as in our case. We therefore provide an atomically resolved high-angle annular dark-field (HAADF) image in Figure 2(d) which clearly shows a truncated octahedron. Both images show highly crystalline NPs with well-defined structures. The brightness in the HAADF image should be related to the thickness of the particle and the square of the average atomic number (*Z*^2^). Therefore, in principle, the precipitation of Pt in the shell should be visible with bright shell atoms and dimmer core atoms. However, as will be shown below, the Pt-shell of PtFe_1.2_ is only 1 ML; the number of shell atoms that contribute to the image is much smaller than the number of atoms in the inner parts, and the atoms on the shell experience much larger thermal vibration than those in the inner part. With these factors combined, the HAADF image does not show any significant brightness contrast between the shell atoms and the core atoms^15^.

**Figure 3:**
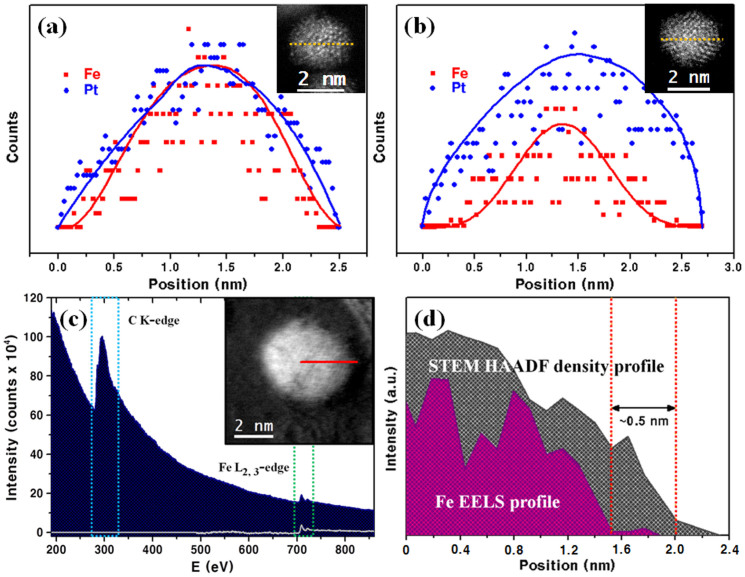
Microscopic analysis results: high-resolution energy-dispersive spectroscopy (HREDS) line mapping images for (a) a PtFe_1.2_ NP and (b) a PtFe_0.4_ NP. (c) Electron energy loss spectroscopy (EELS) of Fe elements in a 4 nm-sized PtFe_1.2_ NP shown in inset and (d) Fe EELS profile overlapped with the NP' STEM-HAADF density profile.

The elemental distributions of PtFe_x_ NPs were investigated by high-resolution EDS (HREDS) equipped on a STEM. In Figures 3(a–b), we compare the HREDS elemental profiles of the NPs of the PtFe_1.2_ and PtFe_0.4_ samples, both of which are 2.3 nm in diameter. The measurements were conducted by scanning a 0.8-Å-sized electron beam along the yellow lines of the NPs, shown in the insets, with 0.2–0.3 Å of gaps between data points. From these plots, the shell thickness is estimated to be 0.2 nm for a PtFe_1.2_ NP and 0.5 nm for PtFe_0.4_. Because the thickness of a monolayer of Pt is about 0.24 nm, these correspond to 1 ML and 2 ML of Pt-shells, respectively. These geometric features of the NPs are verified to be realistic by simplistic model calculations of the on-spherical NPs (see Supplementary Information, Figure S5). By taking into account the densities of Fe and Pt, the Pt-shell of a 2.3 nm PtFe_1.2_ NP is calculated to be 0.25 nm and that of a 2.3 nm PtFe_0.4_ NP is 0.45 nm.

The Pt shell thickness of a PtFe_1.2_ NP (4 nm in diameter) was further confirmed by electron energy loss spectroscopy (EELS). The Pt shell thickness of the PtFe_1.2_ NPs was determined by the Fe EELS line profile combined with the density profile of the NPs from STEM-HAADF. As shown in Figure 3(c), the EELS obtained by measuring the NP along the red line of the inset image (from centre to edge of NP) with a beam of 0.8 Å in size shows a signal of Fe L_2,3_-edge at around 700 eV. In the combined plot of the STEM-HAADF and EELS (Figure 2(d)), the HAADF profile is displaced from the Fe EELS profile by ~0.5 nm, which is the Pt-shell thickness of this NP (Figure 3(d)). The composition and the shell thickness of this NP also agree well with the model calculation shown in Figure S5 (see Supplementary Information). These results clearly show that the PtFe_x_ NPs are formed as Fe-core and Pt-shell structures and that their shell thickness can be controlled from 1 ML to 2 ML.

Electrochemical analyses were carried out using the rotating disk electrode (RDE) technique in a 0.1 M HClO_4_ electrolyte at room temperature (293 K). All of the data set for the PtFe_0.4_/C samples and the reference Pt/C were recorded after cleaning the electrode electrochemically (0–0.6 V, 50 cycles with 500 mV s^−1^) to remove organic residues. Cyclovoltammograms (CVs) of the electrocatalysts are summarised in Figure S6 (see Supplementary Information). Pt/C is composed of ~2.66 nm Pt NPs supported on Vulcan carbon with 37.7 wt%, whereas the loaded amounts of metal contents in PtFe_x_/C are 24–27 wt% (see Supplementary Information, Table S1). The electrochemically-active surface areas (ESAs) of the electrocatalysts were estimated from the CV curves between 0.0 and 0.4 V, with reference to the literature value of 210 μC·cm^−2^ for polycrystalline Pt^28^. The CV curves show the peaks for the surface oxidation and reduction at around 0.7 V. These peaks of PtFe_x_/C are shifted to higher potentials than the respective peaks of Pt/C, indicating that the PtFe_x_/C NPs are less oxophilic than the Pt NPs. Within the PtFe_x_/C samples, as the Fe content (x) increases, the magnitude of the peak shift increases, and hence, the NPs become more oxophilic. According to the kinetics studies in the literature, dissociation of oxygenated species such as O and OH is the rate limiting step of the ORR^9^,^10^,^11^,^29^. Thus, one can expect that the less oxophilic PtFe_x_/C NPs can release the oxygenated species more easily and refresh the catalyst surface more quickly than Pt/C can, resulting in enhanced ORR activity.

**Figure 4:**
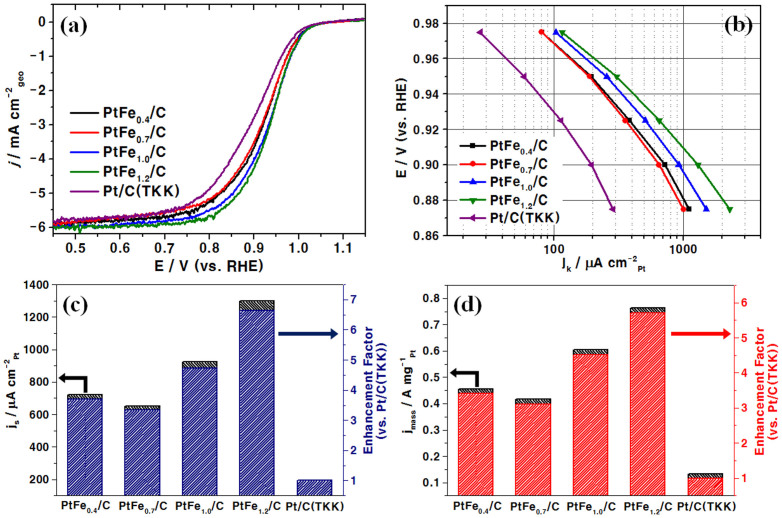
Electrochemical analysis results: (a) Linear sweep voltammograms (LSVs) and (b) Tafel plots of the PtFe_x_/C samples and Pt/C measured in an O_2_-saturated 0.1 M HClO_4_ electrolyte. (c) Specific activity (j_k_, kinetic current density) and (d) specific mass activity (j_mass_) estimated from the LSVs. The j_k_ and j_mass_ data are normalised by the electrochemical surface areas (ESAs) obtained by integration of the H_upd_ region in cyclovoltammograms (CVs).

The linear sweep voltammograms (LSVs), recorded in an O_2_-saturated 0.1 M HClO_4_ electrolyte at a sweep rate of 5 mV s^−1^ (Figure 4(a)), show coherent results with the expectations based on the CV data. The Fe-richest PtFe_1.2_/C shows a considerably higher onset potential at 1.02 V, followed by the other PtFe_x_/C samples with onset potentials at around 1.00 V, than Pt/C at 0.98 V. In particular, the slopes of the curves in the kinetic control region (0.8–1.0 V) of PtFe_x_/C are considerably steeper than that of Pt, indicating greatly improved kinetics. The Tafel plots of the specific activity (j_k_) versus measured potential (Figure 4(b)) clearly show that the ORR kinetics of PtFe_x_/C is definitely superior to that of Pt/C in the high potential range (0.88–0.98 V). In this plot, the electrocatalytic ORR activities are normalised by ESAs and by the masses of Pt used for better comparison. The degree of enhancement of the electrocatalytic ORR activities of PtFe_x_/C appears to be related to the shell thickness of the NPs. From the normalised LSV data, specific activities and specific mass activities (j_mass_) at 0.90 V, are compared in Figures 4(c) and (d), respectively. As shown in Figure 4(c), the specific ORR activity of PtFe_1.2_/C (1298 μA cm^−2^_Pt_) is ~6.7 times larger than that of Pt/C (195 μA cm^−2^_Pt_). The other PtFe_x_/C samples also show enhanced specific activities by 3–5 times. As for the specific mass activity, the value of which is directly correlated with the capacity of the catalysts, PtFe_1.2_/C again shows a drastically enhanced value of 0.765 A mg_Pt_^−1^ (~6 times larger than that of Pt/C).

**Figure 5:**
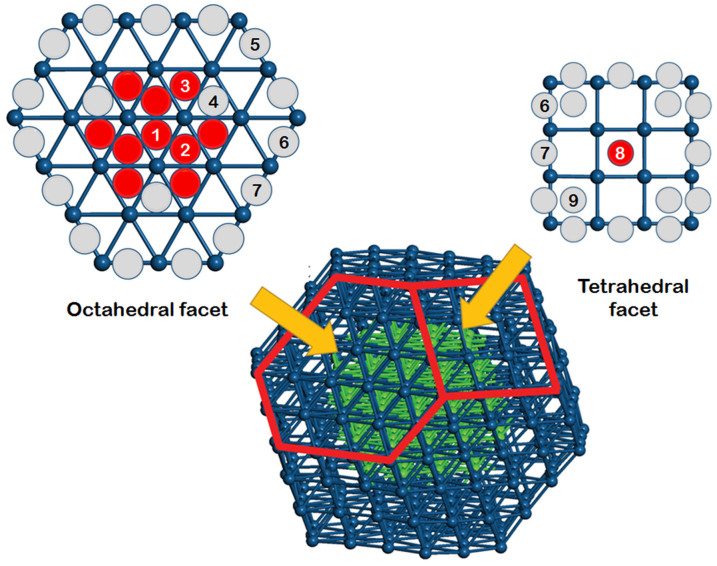
Schematic drawings of facets in a truncated octahedron of Fe@Pt-*n*, where *n* is the number of layers in the Pt shell (blue spheres) on the Fe core (green spheres). Red (grey) circles in the facets represent the sites in which the binding energy to oxygen is weaker (stronger) in Fe@Pt-1 than in Fe@Pt-2.

In order to verify the experimentally-determined trend of the ORR activity with varied Pt shell thickness, we carried out density functional theory (DFT) calculations for the Fe@Pt-*n* NP structures (*n* = 1, 2), where *n* is the number of Pt layers in the shell. Based on the TEM and HAADF-STEM observations shown in Figure 2, we considered the majority of truncated octahedral NP with total of 406 atoms, as shown in Figure 5. All coordinates of the structure were fully relaxed until the Hellmann-Feynman forces were found to be lower than 0.03 eV Å^−1^. The diameters of Fe@Pt-1 and Fe@Pt-2 in this atomistic structural model are about 2.13 and 2.23 nm, respectively. Therefore, Fe@Pt-1 corresponds to Fe_1.2_@Pt and Fe@Pt-2 to Fe_0.4_@Pt in both the size and the elemental distribution. In the relaxed structures, the Pt-Pt bond distances of the outermost Pt shells are 2.63–2.67 Å for Fe@Pt-1 and 2.68–2.72 Å for Fe@Pt-2. The Fe-Fe bond distance in the bulk Fe is about 2.4 Å, while the Pt-Pt bond distance in the bulk Pt is about 2.77 Å^30^. Therefore, the Pt shells are under compressive stress in both structures. This compressive stress is mainly attributable to the lattice mismatch at the interface between the Pt shell and the Fe core. In the case of Fe@Pt-1, the effect of the compressive stress is directly reflected in the significantly reduced Pt-Pt bond distances. In contrast, the effect of the compressive stress to the outermost Pt shell in Fe@Pt-2 is heavily attenuated since the intervening inner Pt shell releases the stress considerably. These theoretical observations of the Pt-Pt bond distances agree well with the experimental data provided in Table 1.

Next, we considered possible binding sites of an oxygen atom on the Fe@Pt-*n* structures, as shown in Figure 5. For each site, we compared the oxygen binding energies between Fe@Pt-1 and Fe@Pt-2 structures. The oxygen binding energy is defined as E_b_ = (E_cluster_ + E_O_) − E_O/cluster_, where E_cluster_, E_O_ and E_O/cluster_ represent total energies of the Fe@Pt-*n* NP, a single O atom and the O-adsorbed Fe@Pt-*n* NP, respectively. The calculated E_b_ values are given in Table S2 (see Supplementary Information). Our results demonstrate that E_b_ strongly depends on the binding site. The edge sites between two facets (5, 6 and 7 in Figure 5) are the strongest binding sites for both Fe@Pt-*n* structures. The binding energies of these sites are too large to allow easy dissociation of the adsorbed oxygen atom. Hence, they do not contribute to the catalytic activity. The terraces of the hexagonal (1, 2, 3 and 4) and the tetragonal facets (8) have weaker binding sites, compared to the edge sites. Since the rate-determining step of ORR is the dissociation of O or OH from the catalyst, these are the probable sites for the catalytic activity of Fe@Pt-*n*. Therefore, we compared the oxygen binding energies of the terrace sites between the two Fe@Pt-*n* structures to assess their relative catalytic activities. As summarised in Table S2, most of the terrace sites have weaker binding of the O atom in Fe@Pt-1 than in Fe@Pt-2. These sites are represented as red circles in Figure 5; the grey circles show the opposite trend. From these results, we can conclude that, comparing the catalytically active sites, the Fe@Pt-1 structure has more weak oxygen binding sites than does Fe@Pt-2.

Mavrikakis *et al.* showed that the binding energy of atomic oxygen is linearly correlated with the binding energy of the transition state of O_2_ dissociation on close-packed transition metal surfaces^31^. Consequently, the O_2_ dissociation kinetics can be estimated from the binding energy of atomic oxygen alone. Therefore, the weaker binding of O on Fe@Pt-1 than on Fe@Pt-2 implies a higher ORR performance of Fe@Pt-1 than of Fe@Pt-2. We thus conclude that these theoretical results are in good agreement with our experimental results.

Catalytic durability is one of the key factors in PEMFC electrode applications. We have carried out the ORR measurements for the most catalytically active PtFe_1.2_/C sample before and after a 5000 cycling test in the potential range from 0.3 to 0.9 V at room temperature in a 0.1 M HClO_4_ solution. The CVs and LSVs before and after the potential cycles (see Supplementary Information, Figures S7(a) and (b), respectively) show negligible changes after the test. In addition, TEM micrographs of the sample indicate an insignificant change of size of NPs after the cycling test (see Supplementary Information, Figure S8). The ORR specific and mass activity are decreased by ~12% and ~18%, respectively (Figure S7(c)) Considering that ~30–40% loss of activity is commonly observed for Pt/C^6^,^32^,^33^, PtFe_1.2_/C demonstrates significantly improved durability. The durability test data strongly indicate that the NPs are completely covered by Pt shells, which function as protection layers for the Fe cores.

## Discussion

We have synthesised a series of Fe*_x_*@Pt core-shell NPs. The syntheses have been designed to take advantage of the characteristics of the newly developed UPS reaction method so that the products form a series of Fe*_x_*@Pt NPs with the Pt-shell thickness systematically varied from 1 ML to 2 ML while the size of the whole NPs remains constant. In the course of this synthesis endeavour, we also obtained experimental data that sheds light on the detailed reaction mechanism of the UPS reaction. The ICP, XRD, TEM, STEM, EDS and EELS data prove that these NPs have highly crystalline core-shell structures and that the Pt-shell thickness can be varied in a controlled fashion. These NPs make it possible to investigate the effect of the shell thickness, free from other effects. Furthermore, our method produced the highest record Fe content (55%) in a core-shell NP. The PtFe*_x_*/C NPs show not only enhanced electrocatalytic activity for the ORR but also superiority in durability. Experimental and theoretical results show that PtFe_1.2_/C with the highest Fe content and 1 ML of Pt shell has the highest ORR activity.

## Methods

### Sample preparation

Fe*_x_*@Pt NPs supported on carbon were prepared by ultrasound-assisted polyol synthesis (UPS). A dispersion of Pt(acac)2 (Aldrich) and Fe(acac)3 (Aldrich) in EG (30 mL) with porous carbon (Ketjen Black) added was irradiated with ultrasound by a high-intensity ultrasonic probe (Sonic and Materials, model VC-500, amplitude 30%, 13 mm solid probe, 20 kHz) for 3 h under Ar. The amount of metal precursors was varied from 0.034 to 0.086 mmol for Fe(acac)3 and from 0.085 to 0.072 mmol for Pt(acac)2. The reaction temperature of 150°C was reached within 30 minute after turning the ultrasound irradiation on and was maintained unchanged without external heating. The dark slurry was filtered, washed with ethanol several times and dried under a vacuum.

### Electrochemical analysis

The electrochemical measurements were carried out by a rotating disk electrode (RDE) technique with a standard three-electrode electrochemical cell. 5 mg of an electrocatalyst was dispersed in 2.5 g of de-ionised water by sonication. 5 μL of the suspension was dropped onto a working electrode (glassy carbon, d = 3 mm), dried in air and 5 μL of a 0.05 wt% Nafion solution was applied. The working electrode was cleaned electrochemically before each measurement. A commercial electrocatalyst composed of Pt NPs on a carbon support (37.7 wt% Pt/C, TKK) was used as a reference.

Cyclic voltammograms (CVs) were recorded in a 0.1 M HClO_4_ aqueous solution at a scan rate of 50 mV s^−1^. An electrochemically active surface area (ECA) of the electrocatalyst was obtained by integrating the CV curve for the hydrogen active region and calibrating with the value of 210 μC cm^−2^ for polycrystalline Pt. The electrocatalytic activity for the ORR was evaluated by using linear sweep voltammetry in 30 min-O_2_ saturated electrolyte (0.1 M HClO_4_) at a scan rate of 5 mV s^−1^ with 1600 rpm. A Ag/AgCl/sat'd KCl reference electrode and a Pt net counter electrode were used in all electrochemical experiments.

### Sample characterisation

X-ray diffraction (XRD) patterns of the samples were recorded by using an Ultima IV X-ray diffractometer (Rigaku, CuKα, *λ* = 0.15406 nm). High-resolution transmission electron microscopy (TEM) imaging and energy dispersive spectroscopy (EDS) measurements were carried out by using a JEM-2100F transmission electron microscope (JEOL). Scanning TEM high-angle annular dark-field (STEM-HAADF) images and EDS mapping profiles were observed by using a JEM-ARM200F model (JEOL) with 0.8 Å of beam size at 200 kV of acceleration voltage equipped with a probe aberration corrector. Electron energy loss spectroscopy (EELS) profiles of the samples were recorded by an EELS attached onto the STEM. Elemental composition analysis of the samples was conducted by using inductively coupled plasma-atomic emission spectrometry (ICP-AES, OPTIMA 4300DV, Perkin-Elmer). UV-vis. spectra were recorded by using a UV/VIS/NIR spectrophotometer (UV-3600, SHIMADZU).

### Computational calculations

Total energy calculations and atomic structural optimisation were performed within the Kohn-Sham DFT with generalised gradient approximation^34^, implemented in the Vienna ab initio simulation package (VASP)^35^,^36^,^37^. The wave functions were expanded using plane waves with a cut-off energy of 400 eV, while the ions were represented by the projector-augmented wave potentials^38^. For the Brillouin-zone integration, only one k-point (k = 0) was chosen^39^.

## Author Contributions

J.H.J. and E.L. performed the experiments, J.P. and G.K. performed the theoretical calculations, and S.H. and Y.U.K. wrote the main manuscript text.

## Supplementary Material

Supplementary Information Supplementray Information

## Figures and Tables

**Table 1:** Characteristics of PtFe_x_/C electrocatalysts. Crystallite sizes are calculated by Scherrer formula on the (111) peaks of XRD patterns of the electrocatalysts and Averaged particle size are obtained by counting of ~200 particles from TEM images

Sample	Crystallite size (nm)	Particle size (nm)	M-M distance (Å)	Lattice constant (Å)
Pt/C (TKK)	2.66	-	2.79(5)	3.95(3)
PtFe_0.4_/C	2.38	2.3 ± 0.4	2.75(6)	3.89(5)
PtFe_0.7_/C	2.43	2.3 ± 0.4	2.74(8)	3.88(6)
PtFe_1.0_/C	2.46	2.3 ± 0.5	2.73(9)	3.87(3)
PtFe_1.2_/C	2.37	2.3 ± 0.4	2.73(5)	3.86(7)
