# Supplementary material for: Rational syntheses of core-shell Fex@Pt nanoparticles for the study of electrocatalytic oxygen reduction reaction
Source: Sci Rep. 2013 Oct 7;3:2872. doi: 10.1038/srep02872 (PMC3791448; doi:10.1038/srep02872)
Supplement: Supplementary Information — Supplementray Information [file srep02872-s1.doc]

**Supplementary Information for**

**Rational syntheses of core-shell Fe*_x_*@Pt nanoparticles for the study of electrocatalytic oxygen reduction reaction**

Ji-Hoon Jang^1^, Eunjik Lee^1^, Jinwoo Park^2^, Gunn Kim^2^, Suklyun Hong^2^*, and Young-Uk Kwon^1^*

^1^Department of Chemistry, BK- 21 School of Chemical Materials Sciences, SAINT/Center for Human Interface Nano Technology, Sungkyunkwan University, Suwon 440-746, Republic of Korea, ^2^Department of Physics and Graphene Research Institute Sejong University, Seoul 143-747, Republic of Korea

**SI-1. Mechanism of the Formation of Fe_x_@Pt Core-Shell Nanoparticles and the Control of the Pt-shell Thickness by UPS Reaction**

**1. The importance of vapour pressure in sonochemical reactions**

According to the acoustic cavitation mechanism described by Suslick^1^, the energy source of chemical changes in sonochemistry is the implosion of the acoustic cavity. The implosion is reported to generate a very high power (~5000 K) and a short life-time (~10^9^ K s^-1^) energy pulse. This mechanism implies that efficient harvesting of the mechanical energy of sonication into chemical changes requires vaporisation of reagents into the cavity. Molecules outside of the cavities are not susceptible to the acoustic cavitation mechanism but may still be affected indirectly through the heat generated by the sonication. Therefore, it is important to note that the volatility of the metal precursors can be an important control parameter in UPS reactions. In the present system, the vapour pressures of Fe(acac)_3_ and Pt(acac)_2_ are reported to be 2.781^2^ and 0.036 Torr^3^, respectively, at 423 K.

**2. Further verifications of the mechanism**

Based on the mechanism described above, the products of reactions with different Fe(acac)_3_/Pt(acac)_2_ ratios can be predicted. When this ratio is very small (i.e., 0.1), Fe(acac)_3_ will be depleted early, producing a small number of Fe nuclei, which will be finished with Pt-shells. When Fe(acac)_3_ is depleted, no further Fe nucleus can be formed, and thus the number of NPs will be small. In contrast, when the Fe(acac)_3_/Pt(acac)_2_ ratio is very large (i.e., 1.5), the formation of complete Pt-shell becomes difficult, which effectively allows the NPs to grow in size.

Our experimental data on such reactions prove that these expectations are indeed working (Figure S4). The TEM image of the product formed from the reaction of the Fe(acac)_3_/Pt(acac)_2_ = 0.1 condition shows that the number of NPs is much smaller than in the other cases shown in Figure S5. However, the particle sizes are relatively uniform. In contrast, the product formed from the reaction of the Fe(acac)_3_/Pt(acac)_2_ = 1.5 condition shows that the number of NPs is much larger and that the size distribution is less homogeneous.


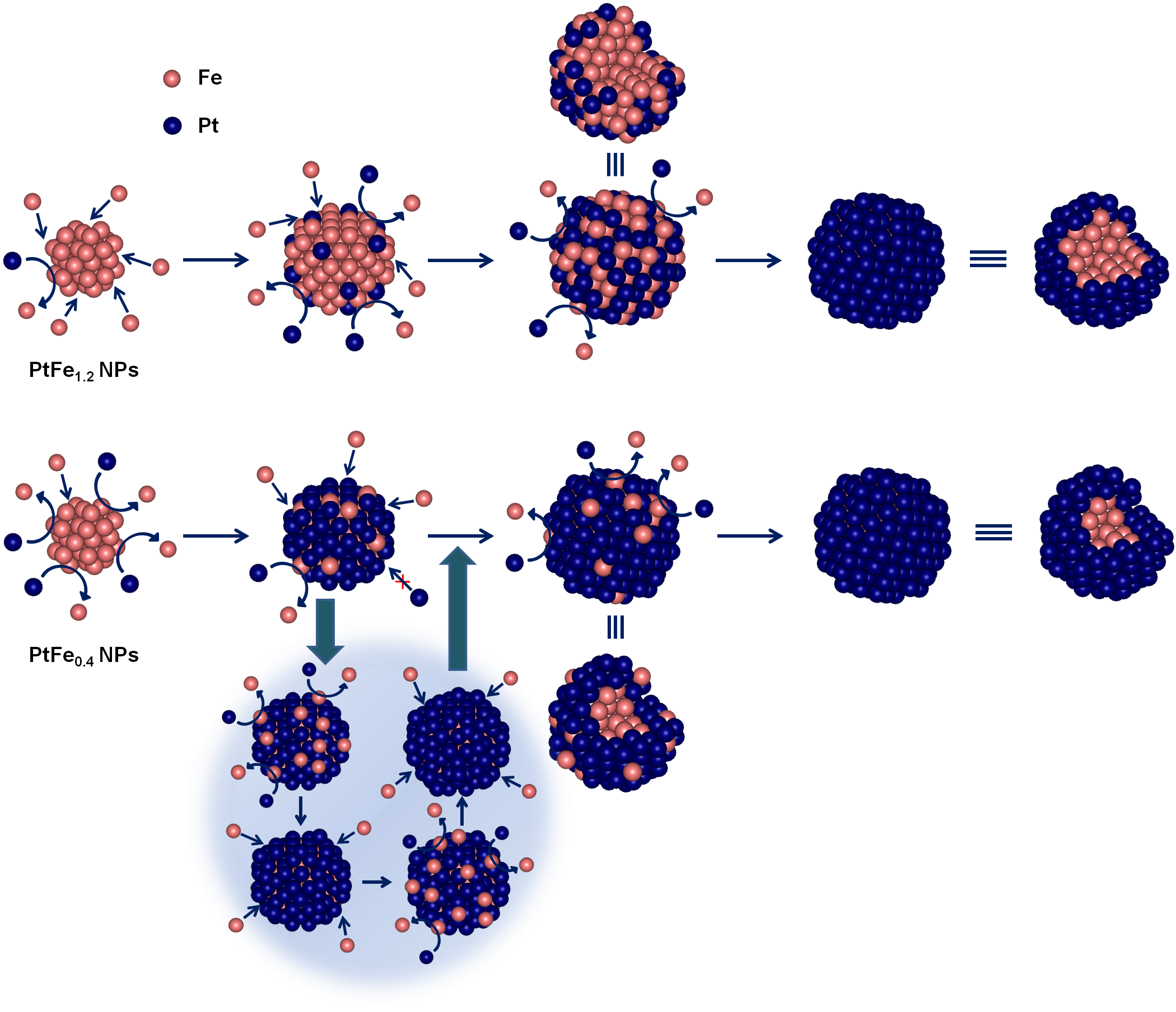


Figure S1. Schematic illustration of the formation mechanism of core-shell PtFe_x_ NPs under two extreme conditions of the reaction rates of depositions of Pt and Fe atoms. If the rate of Fe-addition is faster than that of Pt-addition, the Fe-core will be large and the Pt-shell will be thin (monolayer) (top). If the rate of Pt-addition is faster than that of Fe-addition, the Fe-core will be small and the Pt-shell will be thick (bilayer) (bottom). These cases correspond to PtFe_1.2_ and PtFe_0.4_ NPs, respectively.


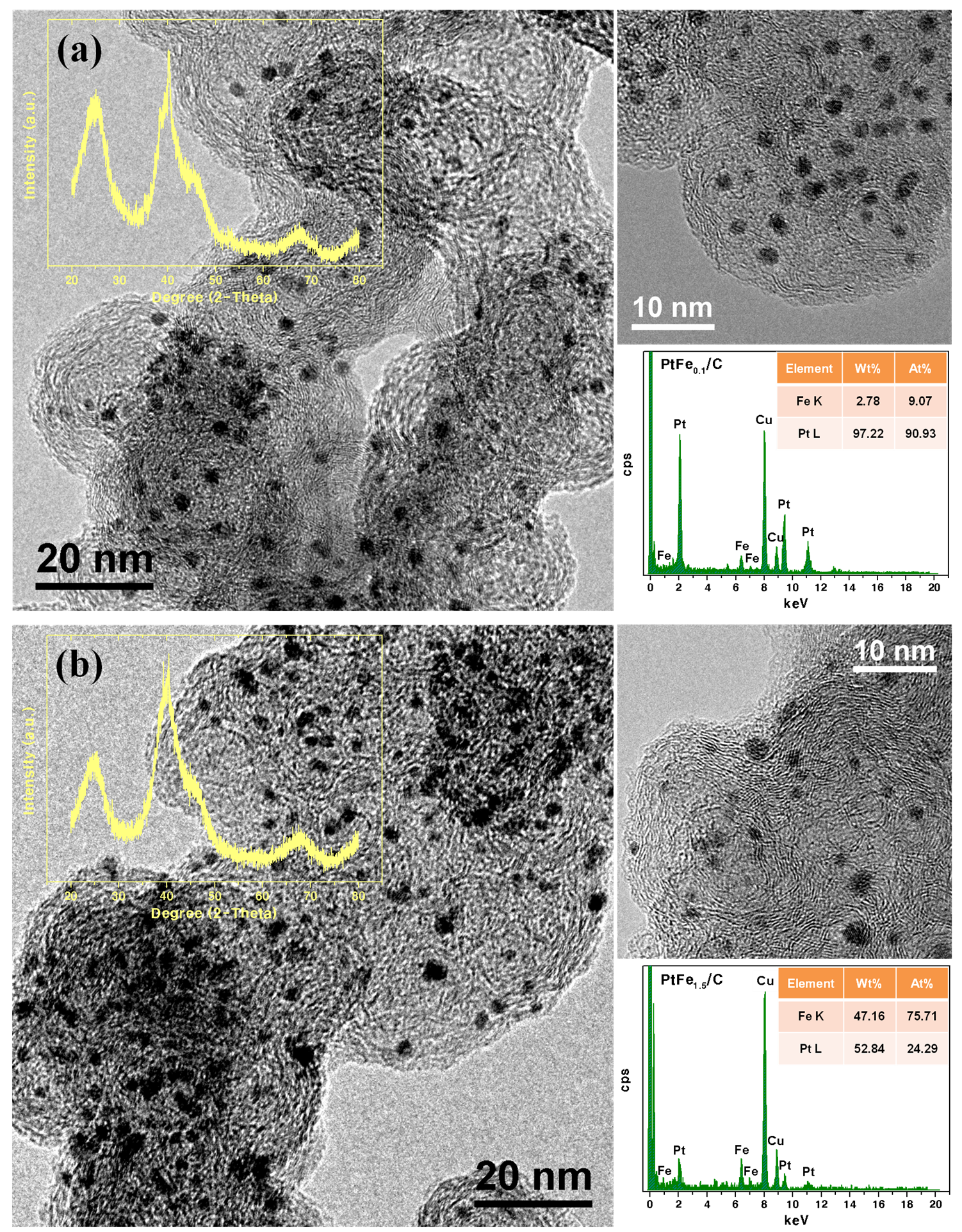


Figure S2. TEM images, XRD patterns, EDS spectra and elemental compositions of individual NPs (marked with dotted red circle on each TEM image) of (a) PtFe_0.1_/C and (b) PtFe_1.5_/C.

**SI-2.** **Additional Characteristics of the Samples**

**Table S1**. Composition and electrochemical characteristics of electrocatalysts.

| **Sample** | **Amount of contents (wt%)^[a]^** | | | **Loaded metal (wt%)^[a]^** | **ECSAs**  **(cm^-2^)^[b]^** | **ECSAs**  **(m^2^ g^-1^)^[b]^** |
| --- | --- | --- | --- | --- | --- | --- |
|  | **Pt** | **Fe** | **C** |  |  |  |
| **Pt/C (TKK)** | 37.7 | 0 | 62.3 | 37.7 | 2.069 | 0.686 |
| **Fe_1.2_@Pt/C** | 20.4 | 6.6 | 73.0 | 27.0 | 0.961 | 0.589 |
| **Fe_1.0_@Pt/C** | 21.3 | 6.3 | 72.4 | 27.6 | 1.114 | 0.654 |
| **Fe_0.7_@Pt/C** | 20.9 | 4.4 | 74.7 | 25.3 | 1.069 | 0.639 |
| **Fe_0.4_@Pt/C** | 21.6 | 3.0 | 75.4 | 24.6 | 1.043 | 0.633 |

^[a]^Obtained from ICP-AES; the content of each element is an averaged value from three separte measurements on each sample.

^[b]^Calculated by integration of H_upd_ regions of CV curves with reference to the literature value of 210 μC·cm^-2^ for polycrystalline Pt.


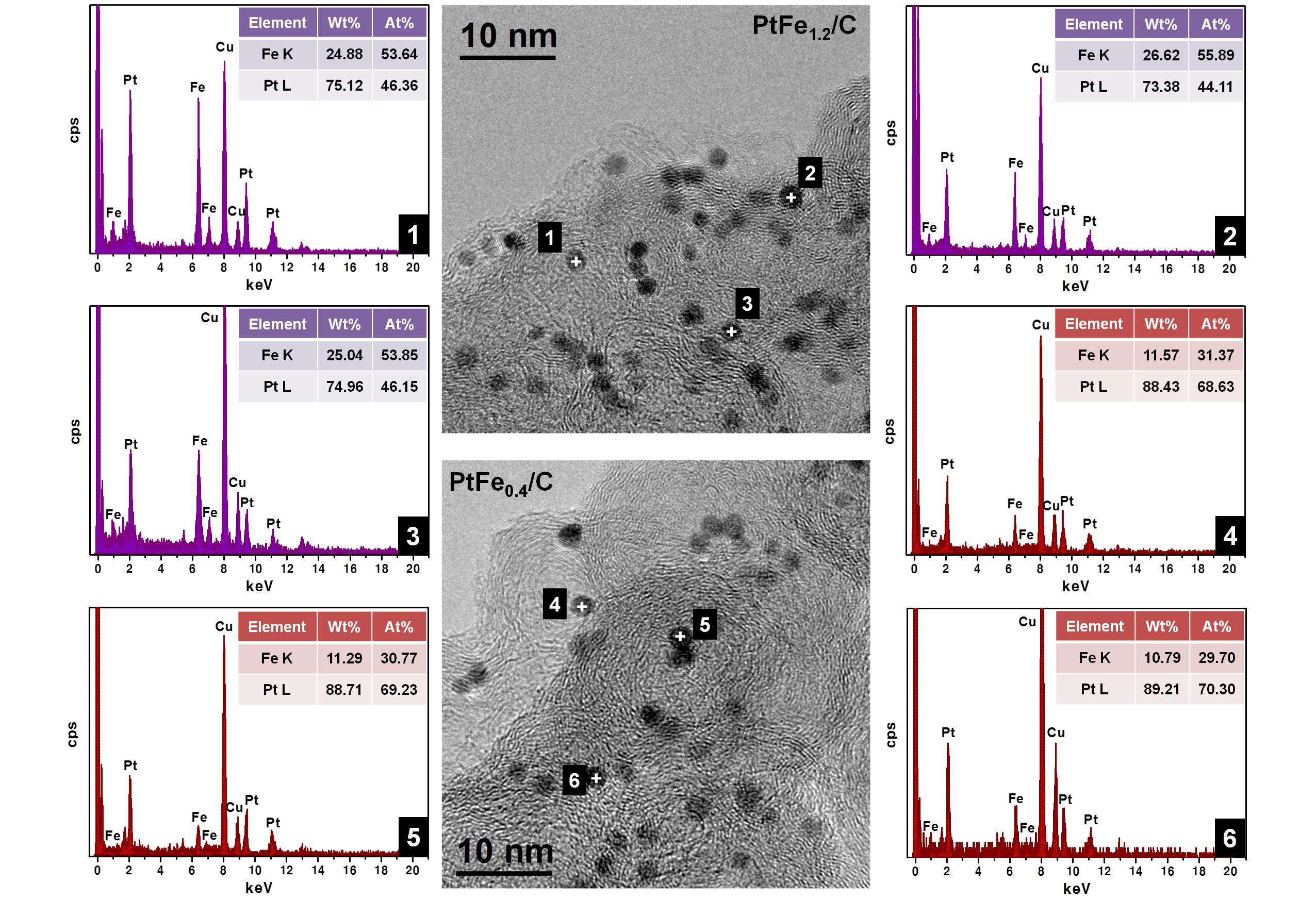


Figure S3. Representative TEM images and the elemental composition of the individual NPs recorded by point EDS of PtFe_1.2_/C and PtFe_0.4_/C


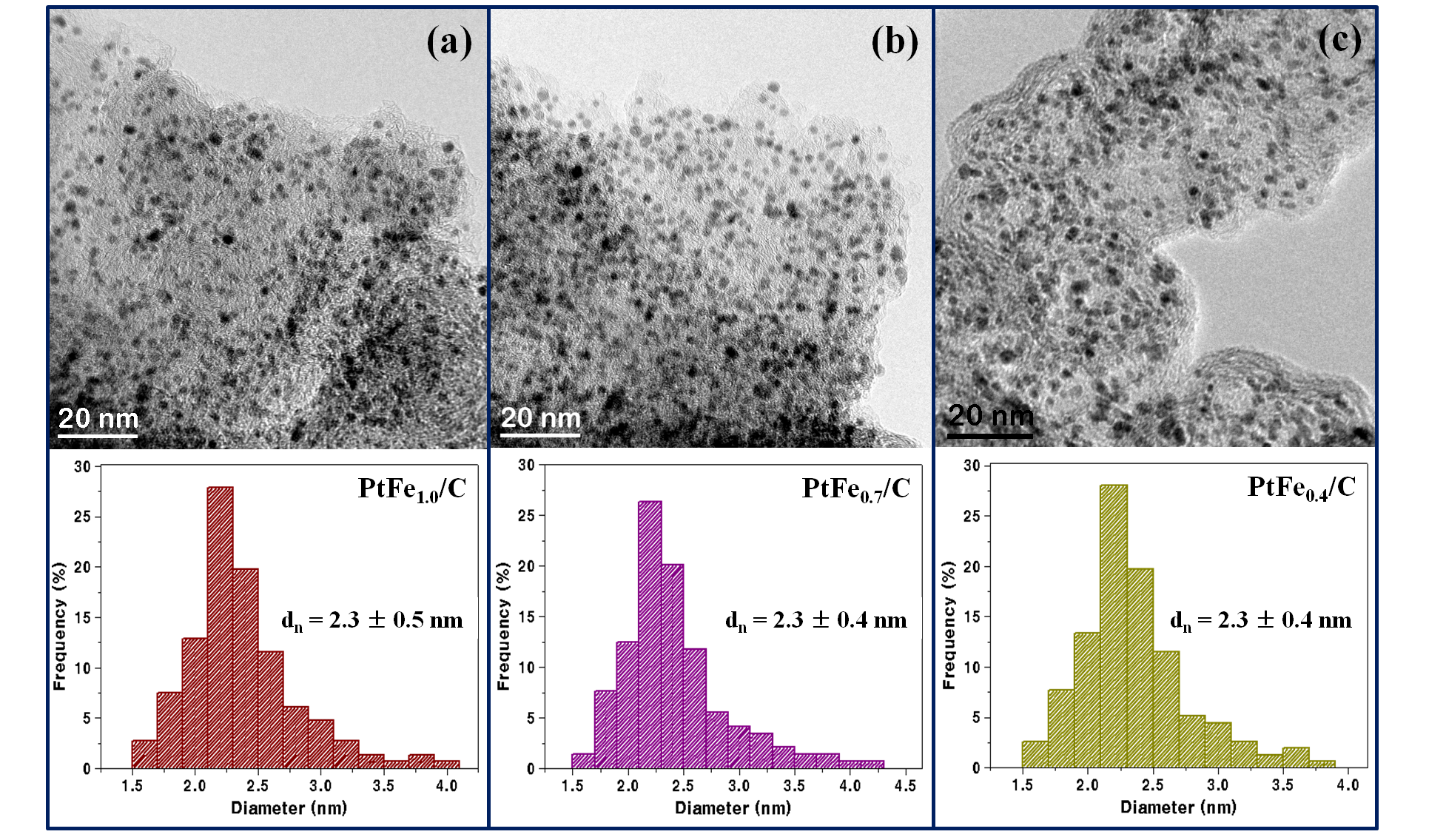


Figure S4. TEM images and size distribution histograms of (a) PtFe_1.0_/C, (b) PtFe_0.7_/C, and (c) PtFe_0.4_/C. The averaged NP’ sizes (d_n_, in diameter) are obtained by counting nearly 250 particles in each sample.


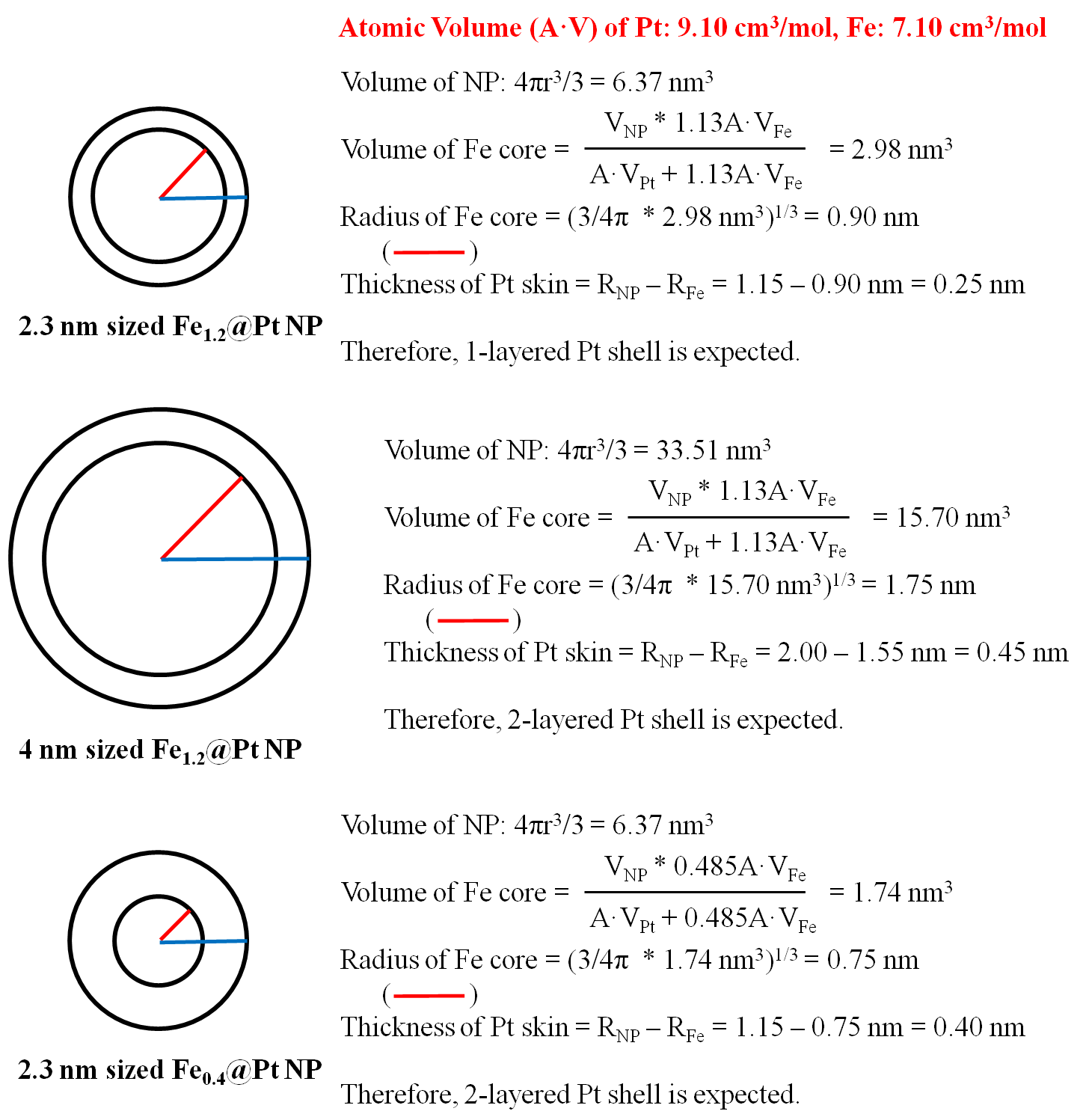


Figure S5. Calculations of the thicknesses of Pt shells based on simplified sphere models of PtFe_1.2_ NP with varied sizes of 2.3 and 4 nm and of 2.3 nm sized PtFe_0.4_ NP.


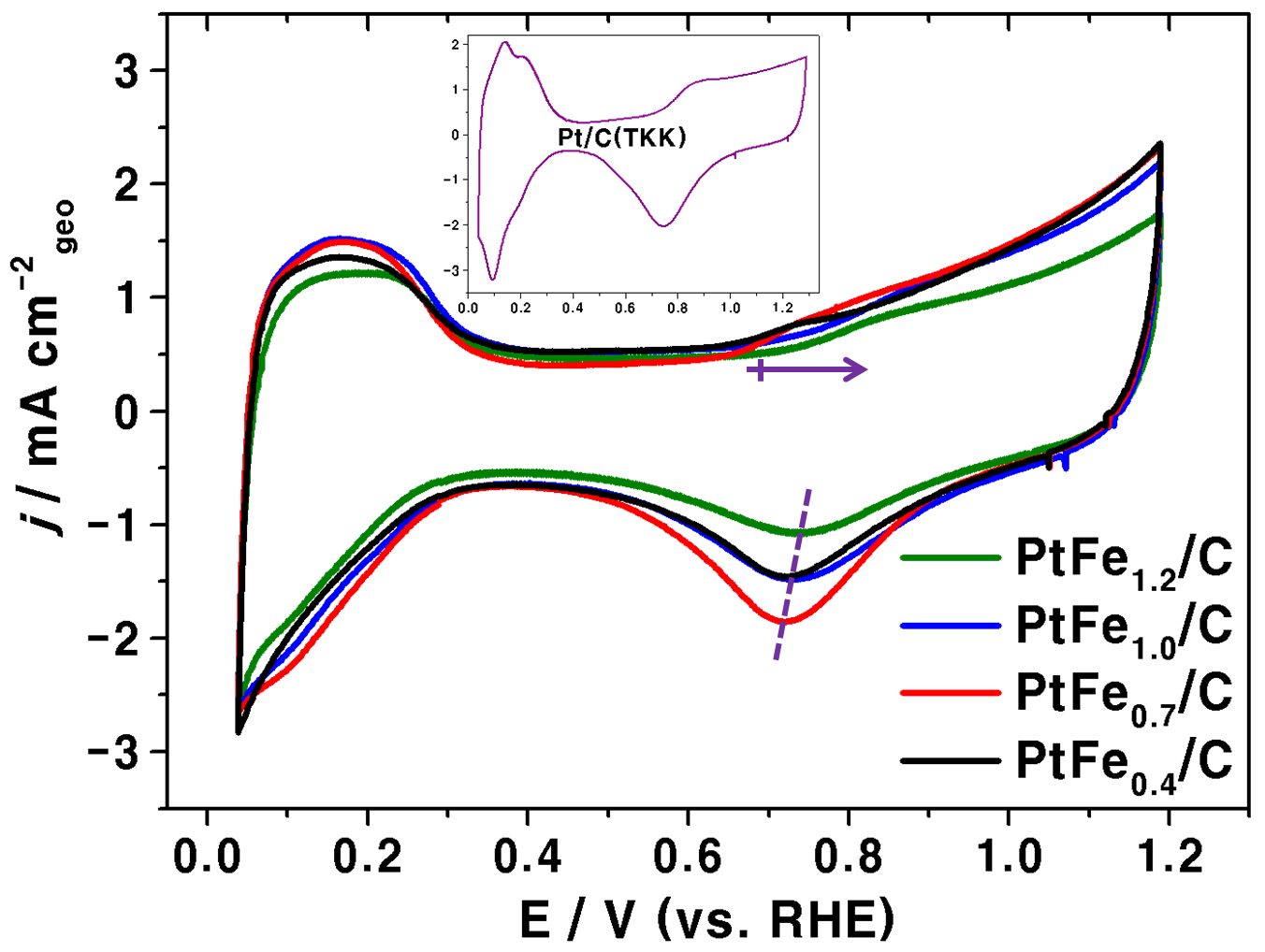


Figure S6. Cyclovoltammograms for Fe*_x_*@Pt/C and Pt/C(TKK) electrocatalysts. The plots are recorded in 0.1 M HClO_4_ electrolyte saturated with N_2_ at room temperature with a scanning rate of 50 mV s^-1^.

**Table S2**. Binding energy (E_b_) calculations of a single O atom on various sites on Fe@Pt-*n* NPs with two different shell thicknesses. The shaded columns are the data on the terrace sites.

|  | | **1** | **2** | **3** | **4** | **5** | **6** | **7** | **8** | **9** |
| --- | --- | --- | --- | --- | --- | --- | --- | --- | --- | --- |
| **^[a]^E_b_ (eV)** | Fe@Pt-1 | 4.77 | 5.01 | 4.99 | 5.76 | 6.90 | 7.01 | 6.73 | 5.65 | 7.11 |
|  | Fe@Pt-2 | 5.08 | 5.37 | 5.35 | 5.63 | 6.31 | 6.41 | 6.46 | 5.67 | 6.08 |
| **^[b]^∆E_b_ (eV)** | | -0.31 | -0.37 | -0.35 | 0.14 | 0.59 | 0.61 | 0.27 | -0.02 | 1.03 |

^[a]^ The binding energy is defined as E_b_ = (E_cluster_ + E_O_) – E_O/cluster_, where E_cluster_, E_O_ and E_O/cluster_ represent total energies of the Fe@Pt-*n* cluster, a single O atom, and the O-adsorbed Fe@Pt-*n* NP, respectively.

^[b]^ **∆**E**_b_**, defined as E_b_(Fe@Pt-1) - E_b_(Fe@Pt-2), represents the difference in binding energy of oxygen between Fe@Pt-1 and Fe@Pt-2.


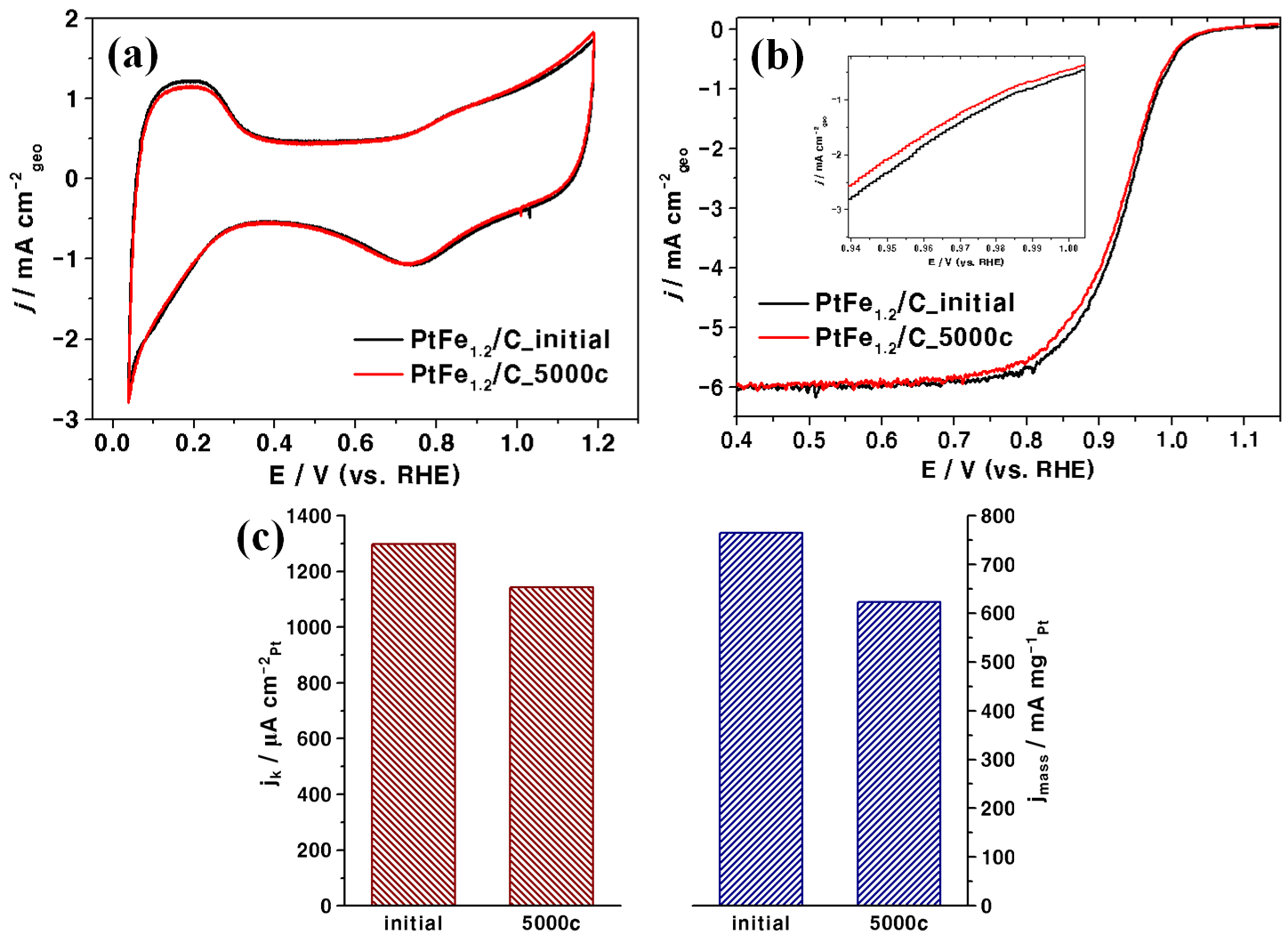


**Figure S7**. Durability test results for PtFe_1.2_/C electrocatalysts: (a) cyclovoltammograms, (b) linear sweep voltammograms (LSVs) for ORR and (c) specific activity (j_k_, kinetic current density) and specific mass activity (j_mass_) estimated from the LSVs. The results were obtained before and after a 5000 cycling test between 0.3 and 0.9 V vs. RHE at room temperature in a 0.1 M HClO_4_ electrolyte.


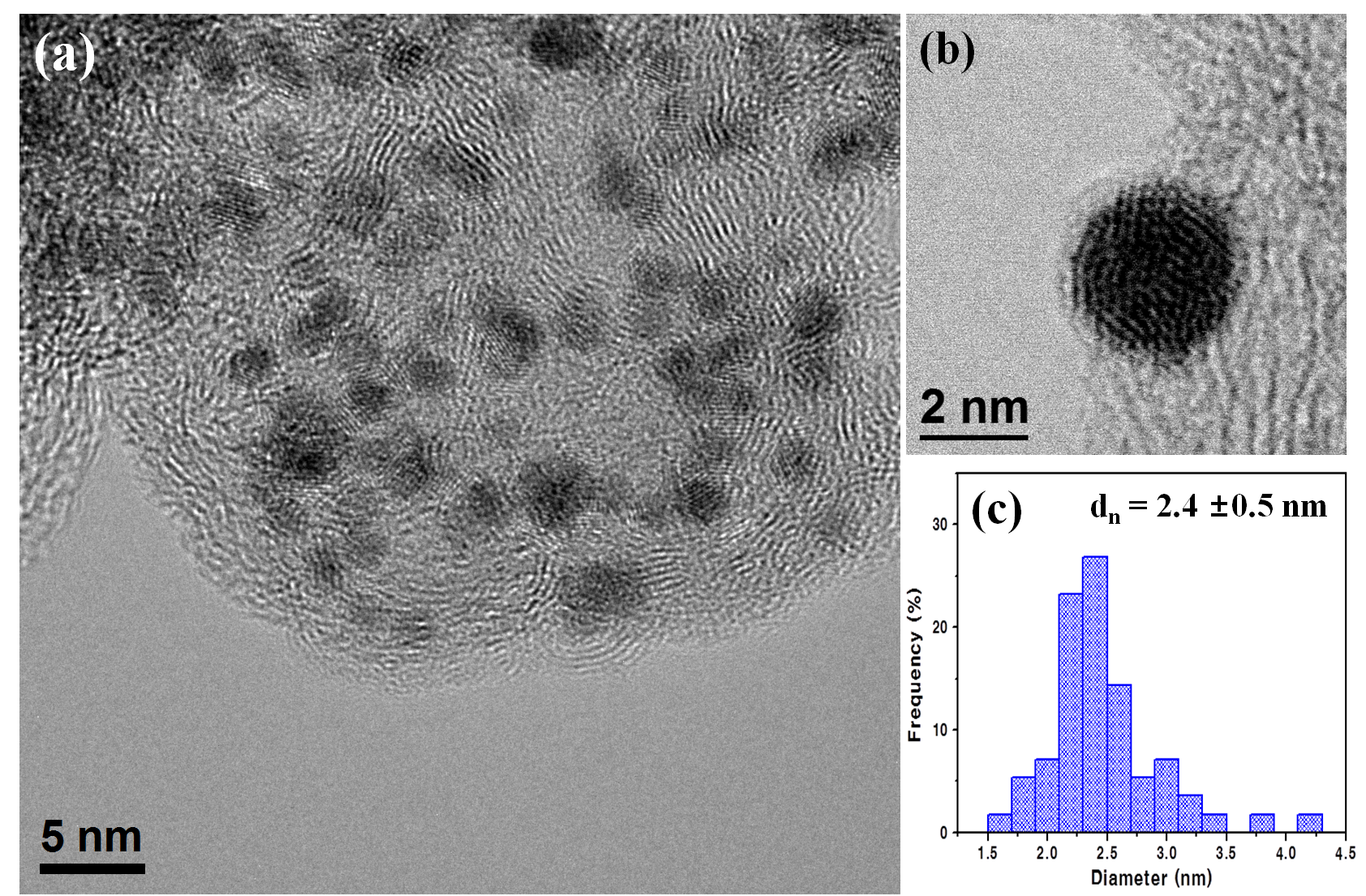


Figure S8. Characterization results of PtFe_1.2_/C electrocatalysts after durability test: (a) TEM micrograph, (b) magnified TEM image and (c) size distribution histogram.

**SI-3. References for Supplementary Information**

1. K. S. Suslick, *Science* **1990**, *247*, 1439-1445.

2. B. D. Fahlman, A. R. Barron, *Adv. Mater. Opt. Electron.* **2000**, *10*, 223-232.

3. N. B. Morozova, G. I. Zharkova, P. P. Semyannikov, S. V. Sysoev, I. K. Igumenov, N. E. Fedotova, N. V. Gelfond, *J. Phys. IV France* **2001**, *11*, 609-616.
